# Locus- and Site-Specific DNA Methylation of 19 kDa Zein Genes in Maize

**DOI:** 10.1371/journal.pone.0146416

**Published:** 2016-01-07

**Authors:** Jian-Hong Xu, Ruixian Wang, Xinxin Li, Mihai Miclaus, Joachim Messing

**Affiliations:** 1 Institute of Crop Science, Zhejiang Key Laboratory of Crop Germplasm, Zhejiang University, Hangzhou, Zhejiang 310058, China; 2 National Institute of Research and Development for Biological Sciences, Cluj-Napoca, Romania; 3 Waksman Institute of Microbiology, Rutgers, The State University of New Jersey, Piscataway, New Jersey, 08854, United States of America; Institute of Crop Sciences, CHINA

## Abstract

An interesting question in maize development is why only a single zein gene is highly expressed in each of the 19-kDa zein gene clusters (A and B types), *z1A2*-1 and *z1B4*, in the immature endosperm. For instance, epigenetic marks could provide a structural difference. Therefore, we investigated the DNA methylation of the arrays of gene copies in both promoter and gene body regions of leaf (non-expressing tissue as a control), normal endosperm, and cultured endosperm. Although we could show that expressed genes have much lower methylation levels in promoter regions than silent ones in both leaf and normal endosperm, there was surprisingly also a difference in the pattern of the *z1A* and *z1B* gene clusters. The expression of *z1B* gene is suppressed by increased DNA methylation and activated with reduced DNA methylation, whereas *z1A* gene expression is not. DNA methylation in gene coding regions is higher in leaf than in endosperm, whereas no significant difference is observed in gene bodies between expressed and non-expressed gene copies. A median CHG methylation (25–30%) appears to be optimal for gene expression. Moreover, tissue-cultured endosperm can reset the DNA methylation pattern and tissue-specific gene expression. These results reveal that DNA methylation changes of the 19-kDa zein genes is subject to plant development and tissue culture treatment, but varies in different chromosomal locations, indicating that DNA methylation changes do not apply to gene expression in a uniform fashion. Because tissue culture is used to produce transgenic plants, these studies provide new insights into variation of gene expression of integrated sequences.

## Introduction

Methylation of chromosomal DNA is a major mark of epigenetic regulation of gene expression, which is present in some fungi and insects, but in all mammals and higher plants [1–4]. It can be divided into three types of CG, CHG, and CHH (H = A, C, or T). In mammals, DNA methylation occurs predominantly in the CG context, whereas it can occur in all three contexts in plants [5]. In general, high DNA methylation within the promoter region can strongly correlate with gene inactivity [6,7]. Most expressed genes in plants have unmethylated promoters, but genes with methylated promoters show a greater degree of tissue-specific expression, as shown by association with methylation changes near transcriptional start sites and promoters [8,9]. Furthermore, DNA methylation is also associated with the formation of epialleles [10]. In addition, DNA methylation of the promoter is also correlated with transgene silencing in plants [11,12].

Besides promoters, gene body methylation has also been associated with transcriptional levels, which is also conserved between plants and animals [13–16]. This type of hypomethylation also correlates to gene expression [17,18], and corresponds between moderate to high level of expression in various tissue types [4,8,16,19], whereas excessive DNA methylation will inhibit gene expression [1]. Highly expressed genes have intermediate gene body methylation in rice [18]. Furthermore, methylated genes are enriched for basal cellular functions as house-keeping functions, such as those involving translation, transcription and organelles [17].

Tissue culture can induce DNA methylation changes and the activation of transposable elements (TEs) [20–23]. In *Arabidopsis*, dramatic hypomethylated cytosines were observed in cell suspension culture with activation of transposition of TEs [24]. Endogenous gene expression could also be activated or inactivated in cultured tissues by altering cytosine methylation. In maize, tissue culture can induce consistent DNA methylation changes [25] and epigenetic somaclonal variation of endogenous genes [26,27]. The *p1-wr* allele of *pericarp color 1* (*p1*) induces red phlobaphenes pigmentation in the cob glumes in maize, showed varying degrees of loss of *p1* function in culture-induced tissue. This alteration is associated with the increased methylation of 3’ region in the second intron of *P1-wr*, resulting almost in the complete loss of transcripts [26].

Maize is one of the most important cereals in the world. It is a major source of reduced nitrogen for livestock and humans, derived from its major seed storage proteins, also known as zeins. They belong to the superfamily of the prolamins, which are rich in proline and glutamine, and poor in lysine [28,29]. Zeins can be divided into four classes according to amino acid sequences: α, β, γ and δ-zein. The α-zeins can further be divided into four subgroups, *z1A*, *z1B*, *z1C*, and *z1D*, based on sequence homology. The *z1A*, *z1B*, and *z1D* subgroups have a relative molecular weight of 19-kDa and *z1C* of 22-kDa and their genes are located in six chromosomal locations. In B73, only two out of 26 of the 19-kDa zein gene copies, *z1A2*-1 and *z1B4*, are expressed at high levels [30]. Another gene, *z1B6*, is also expressed but at a much lower level. In contrast, other gene copies are expressed at very low levels or not at all because they appear to be silenced or due to in-frame stop codons. Expression levels of *z1B* zeins could clearly be changed in tissue culture [30]. However, how this affects all gene-copies remains rather unclear. This question prompted us to investigate DNA methylation of individual zein gene copies in both promoter regions and gene bodies in leaf, normal endosperm, and cultured endosperm. We found that the differential DNA methylation of 19-kDa zein genes in both promoters and gene bodies, and in tissue culture affected locus-specific DNA methylation with different impact in the regulation of zein gene expression.

## Results

### DNA methylation in promoters of 19-kDa zein genes

**Fig 1:**
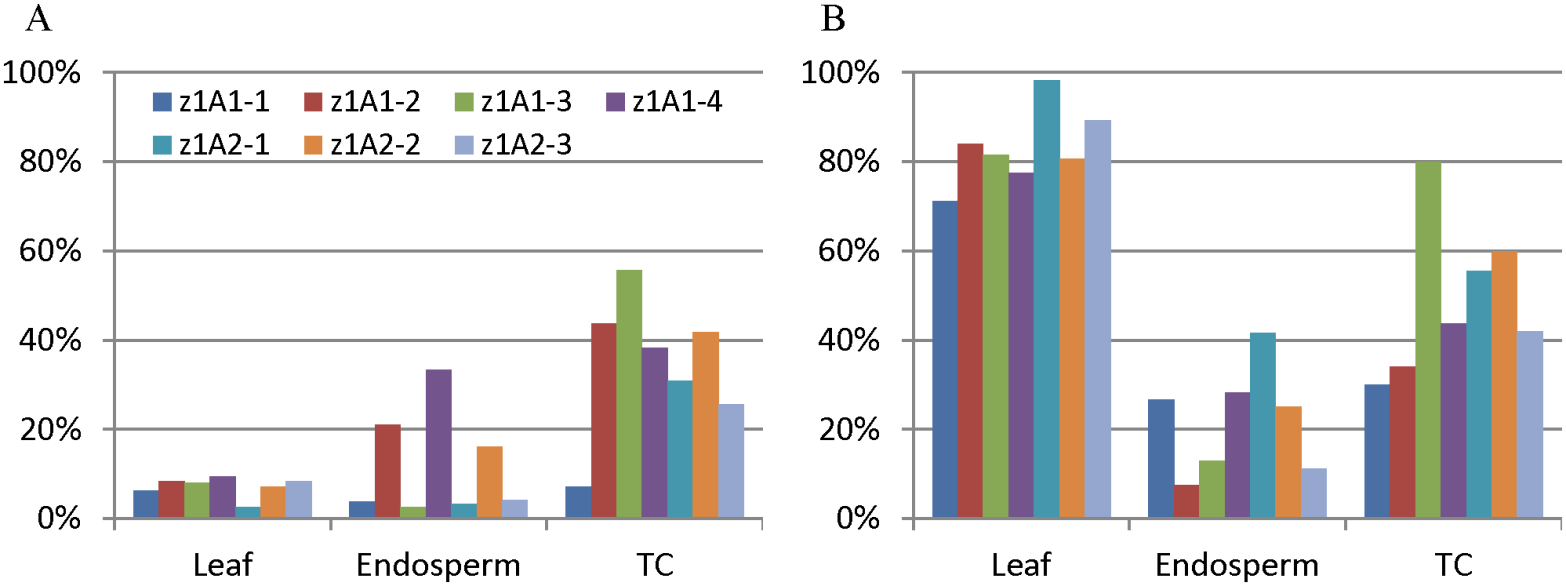
Methylation status of promoter regions of *z1A* genes. Panel (A) displays average DNA methylation, and (B) CG methylation in leaf, endosperm and tissue-cultured endosperm (TC) as graphic bars. The universal primers of *z1A* genes, described in our previous study [30], were used for bisulfite PCR amplification. Ninety-six colonies were sequenced, and each sequence was matched to individual *z1A* genes based on sequence similarity. The average methylation levels were calculated for individual genes with all three contents (A) and CG (B). The color code for individual gene copies is displayed as an insert.

**Fig 2:**
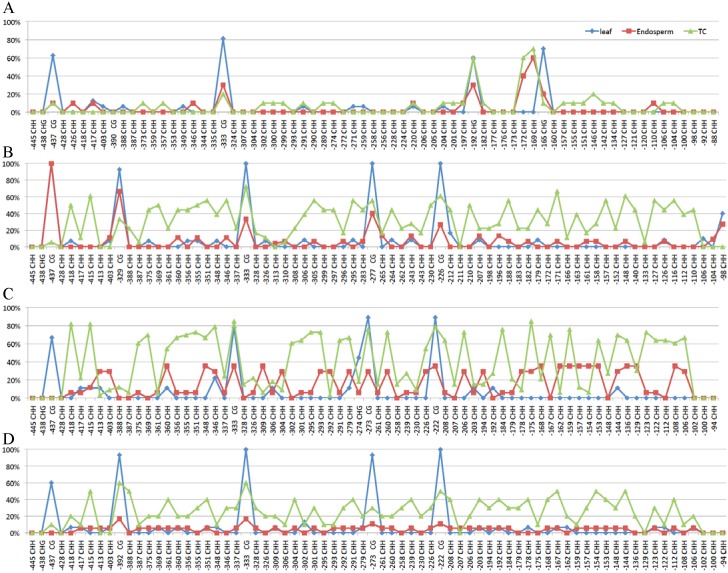
Methylation chromatograms of promoter regions of representative *z1A* genes. The (A) *z1A1*-1, (B) *z1A2*-1, (C) *z1A2*-2, (D) *z1A2*-3 gene in leaf (blue), endosperm (red) and tissue cultured endosperm (TC) (green). The methylation level of specific cytosine in each *z1A* gene was calculated based on its C to T conversion level.

Previous studies showed hypermethylation of five CGs in promoters of *z1A* gene copies in leaf tissue, but not in endosperm, whereas *z1B* copies had moderate DNA methylation (only CHH context) in both leaf and endosperm [30]. Whereas *z1A* comprises 12 genes in two locations on chromosome 4s and *z1B* nine gene copies in one location on chromosome 7s, it was unknown whether each copy has a similar DNA methylation pattern. Therefore, DNA methylation of individual *z1A* and *z1B* gene copies was analyzed by bisulfite sequencing with universal and specific primers (S1 Fig). The average DNA methylation in the promoter region of *z1A* genes was less than 10% in leaf tissue; some of them were even lower than in endosperm (Figs 1A and 2). Four or five CGs were hypermethylated in leaf, much higher than in endosperm (Fig 1B, S1 Table), suggesting that the higher promoter CG methylation could provide tissue specificity in gene expression because of the differential methylation between expressing and non-expressing tissue.

**Fig 3:**
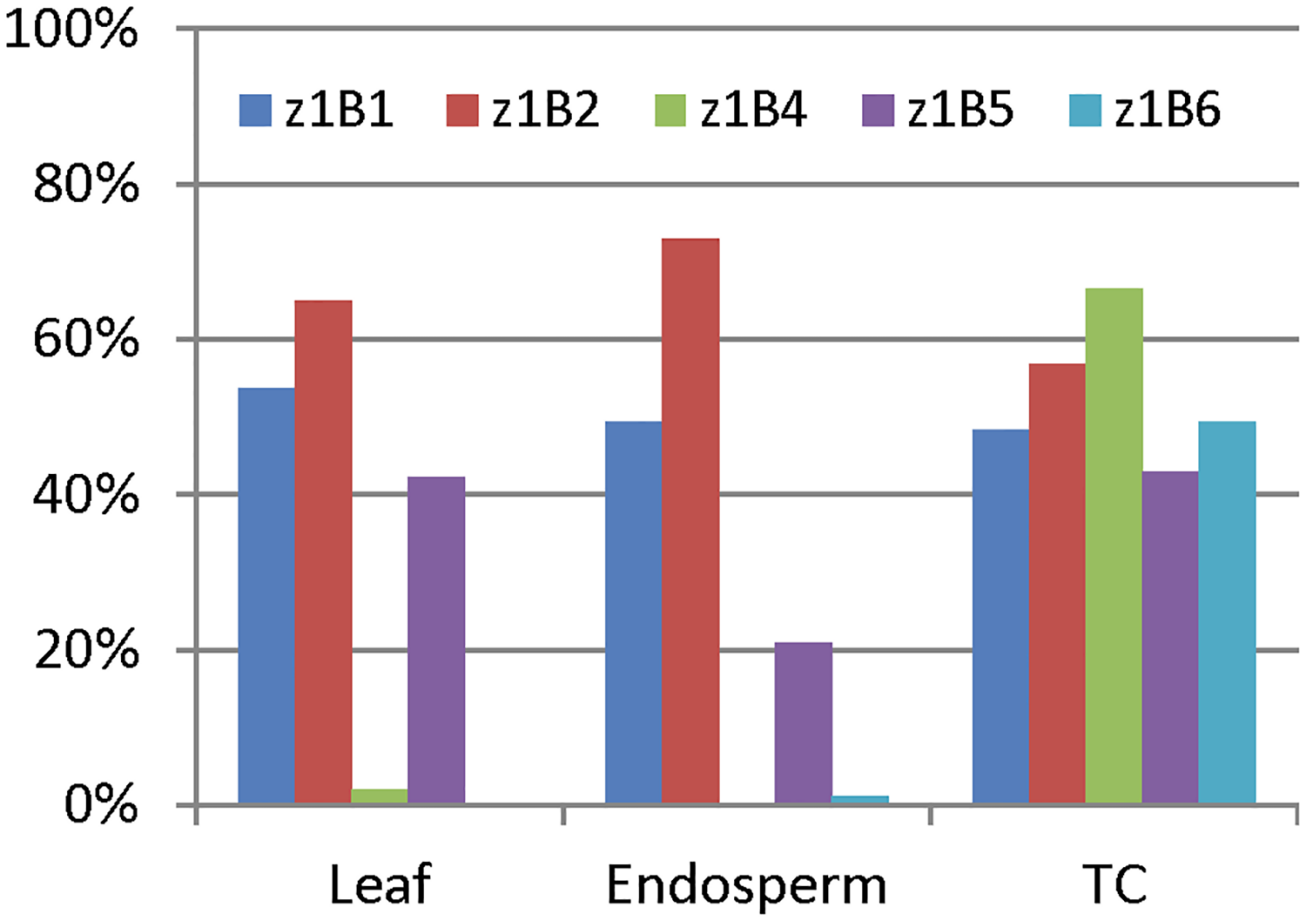
Methylation status of promoter regions of *z1B* genes. The average DNA methylation of *z1B* genes in leaf, endosperm and tissue-cultured endosperm is displayed as bar graphs. The universal primers of *z1B* genes described in our previous study [30] were used for bisulfite PCR amplification. The color code for individual gene copies is displayed as an insert.

**Fig 4:**
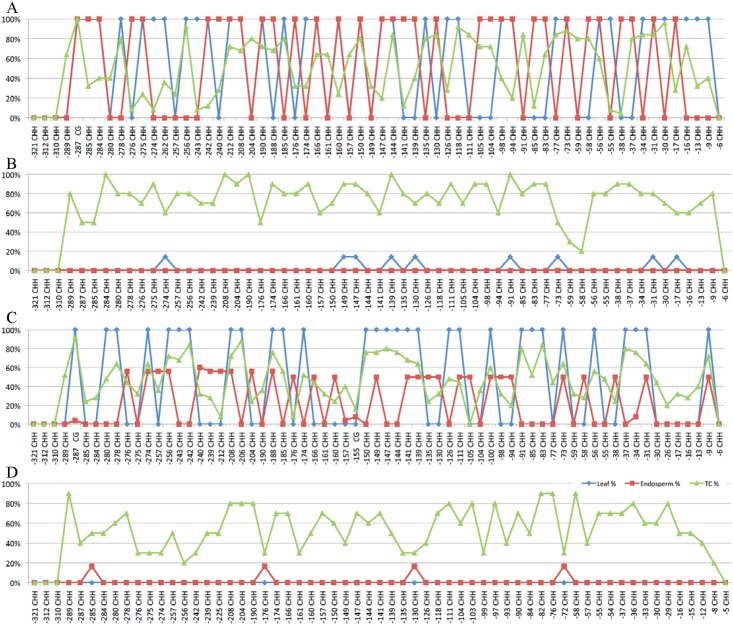
Methylation chromatograms of promoter regions of representative *z1B* genes. (A) *z1B1*, (B) *z1B4*, (C) *z1B5* and (D) *z1B6* in leaf (blue), endosperm (red) and tissue-cultured endosperm (TC) (green).

Such a difference was not found in all *z1B* genes. The two highest expressed gene copies, *z1B4* and *z1B6*, have very low DNA methylation in both leaf and endosperm, whereas all others have higher DNA methylation (up to 65% and 73%) and low level of gene expression (Figs 3 and 4, S1 Table). Notably, the DNA methylation status in *z1B1* is quite interesting; each site was either completely methylated or unmethylated (Fig 4A), although the average DNA methylation levels are similar (60% and 55%) in leaf and endosperm (S1 Table).

### Tissue culture induced locus-specific DNA methylation in 19-kDa zein genes

Gene expression was dramatically reduced for *z1B4* and *z1B6* but enhanced for *z1B1* in cultured endosperm when compared to normal endosperm, whereas that of *z1A* gene copies was not significantly changed [30]. In order to validate whether DNA methylation of promoters can be changed in 19-kDa zein genes during tissue culture, DNA methylation patterns were investigated in cultured endosperm. Surprisingly, promoter DNA methylation of most *z1A* gene copies was significantly increased in cultured endosperm compared to leaf and normal endosperm (P_Leaf vs TC_ = 0.009, P_Endosperm vs TC_ = 0.006), even in the CG context (P_Leaf vs TC_ = 0.011, P_Endosperm vs TC_ = 0.045) (Figs 1 and 2, S1 Table). Although expression of *z1A* gene copies was not changed significantly, it still suggested that DNA demethylation was not required for *z1A* gene expression.

Similarly, DNA methylation of promoter regions of two highly expressed *z1B* genes, *z1B4* and *z1B6*, are increased sharply from 0 to 76.18% and 0.35% to 56.32%, respectively (Figs 3 and 4, S1 Table). However, as this change occurs during the culturing of endosperm cells, the increase in DNA methylation in the respective promoters correlated with reduced expression levels of *z1B4* and *z1B6*, different to the *z1A* loci. Yet, the average DNA methylation was not changed significantly in *z1B1* (S1 Table, 55% in endosperm and 53.93% in cultured endosperm). Further analysis showed that in all 60 cytosines of *z1B1* only 36 were completely methylated in leaf and 33 in endosperm, respectively, and other cytosines were not methylated, whereas all 60 cytosines were partially methylated in cultured endosperm (Fig 4A). Still, the average methylation level of methylated cytosine was decreased from 100% in leaf and endosperm to 53.93% in cultured endosperm, which correlates with a doubling of the *z1B1* gene expression levels in cultured endosperm, presenting a case of activation of a silenced gene. It also suggests a role for gene copies that has been elusive so far because one can envision them as a reserve sink for reduced nitrogen that could be increased with an epigenetic change.

### DNA Methylation patterns of gene body of 19-kDa zein genes

Although the DNA methylation of the promoter could be a critical feature in gene expression, DNA methylation of gene bodies has also been implicated in the regulation of gene expression. As *z1A2*-1 and *z1B4* contribute around 90% of the transcripts of the *z1A* and *z1B* genes, respectively, two *z1A1* (*z1A1*-4 and *z1A1*-5), three *z1A2* genes (*z1A2*-1, *z1A2*-2 and *z1A2*-3), and six *z1B* genes (*z1B1*-*z1B6*) were investigated in respect to DNA methylation in their gene bodies.

Overall, DNA methylation of *z1A* and *z1B* gene bodies is lower in endosperm than in leaf, although the pattern and level were different (Table 1). Both *z1A* and *z1B* genes have hypermethylation of CG (≈ 90%) in leaf, which was reduced by 70% in endosperm, whereas CHG methylation was decreased more than 50% from leaf to endosperm. However, CHG gene body methylation in endosperm ranges from 13.77% to 51.34% in the *z1A*, and from 14.30% to 69.01% in the *z1B* gene cluster (Table 1). Although CHH methylation was increased to about 33% in *z1A* gene bodies, and decreased around 30% in *z1B* gene bodies, it did not correlate with gene expression levels, as methylation levels were low (around 3%). The two highest expressed genes, *z1A2*-1 and *z1B4*, had 28.64% and 26.01% CHG methylation respectively, whereas *z1A2*-2 and *z1B6* had only half the CHG methylation (13.77% and 14.30%) (Table 1), suggesting that an intermediate CHG methylation in coding regions could represent a more stable state for gene expression.

**Table 1:** DNA metylation of z1A and z1B gene bodies.

Zein genes		Leaf				Endosperm			
		CG	CHG	CHH	Clone #	CG	CHG	CHH	Clone #
*z1A*	*z1A1-4*	77.99%	75.60%	2.49%	11	75.86%	35.63%	2.30%	9
	*z1A1-5*	94.21%	75.38%	2.96%	13	82.56%	51.34%	7.71%	20
	*z1A2-1*	80.82%	69.70%	3.29%	9	74.54%	28.64%	2.56%	11
	*z1A2-2*	89.32%	71.64%	3.00%	22	56.64%	13.77%	2.05%	18
	*z1A2-3*	88.85%	62.07%	1.98%	10	62.73%	27.97%	2.42%	14
*z1B*	*z1B1*	83.74%	64.68%	6.99%	10	75.97%	46.73%	4.17%	11
	*z1B2*	94.37%	80.12%	3.71%	20	58.12%	31.76%	3.32%	13
	*z1B3*	97.56%	83.08%	3.91%	13	44.01%	14.80%	2.49%	10
	*z1B4*	95.73%	81.88%	3.90%	15	78.55%	26.01%	2.38%	9
	*z1B5*	99.23%	87.04%	3.21%	9	89.35%	69.01%	4.03%	10
	*z1B6*	82.05%	74.17%	4.75%	16	72.73%	14.30%	2.25%	11

### Methylation status in CCG context

**Fig 5:**
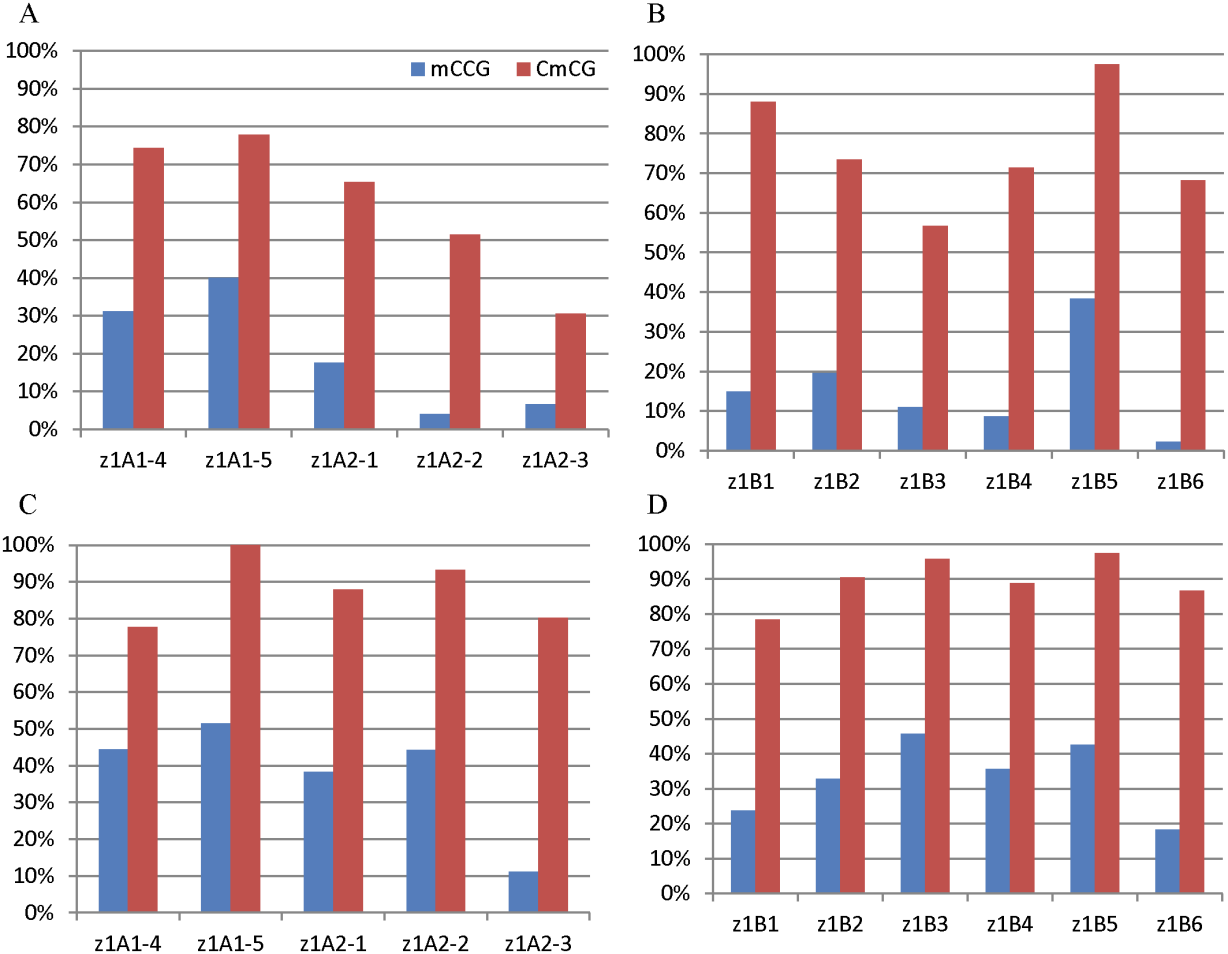
C methylation at CCG sites. Methylation levels of *z1A* (A, C) and *z1B* (B, D) gene coding regions in leaf (C, D) and endosperm (A, B) is shown. The methylation of outer and inner C is marked with mCCG (blue) and CmCG (red), respectively.

CHG represents CAG, CTG and CCG triplets, where CCG has two cytosines that could be methylated simultaneously. As the second cytosine is of the CG type, it has much higher methylation than the first cytosine in CCG [31,32]. We compared the methylation level of the first cytosine to the second cytosine in CCG, which was marked as mCCG and CmCG, respectively. Not surprisingly, mCCG is much lower than CmCG (Fig 5, S2 Table). In *z1A* and *z1B* genes, the average CmCG is 87.81% and 89.62% in leaf, 61.51% and 75.85% in endosperm, respectively, similar to the average CG methylation.

**Fig 6:**
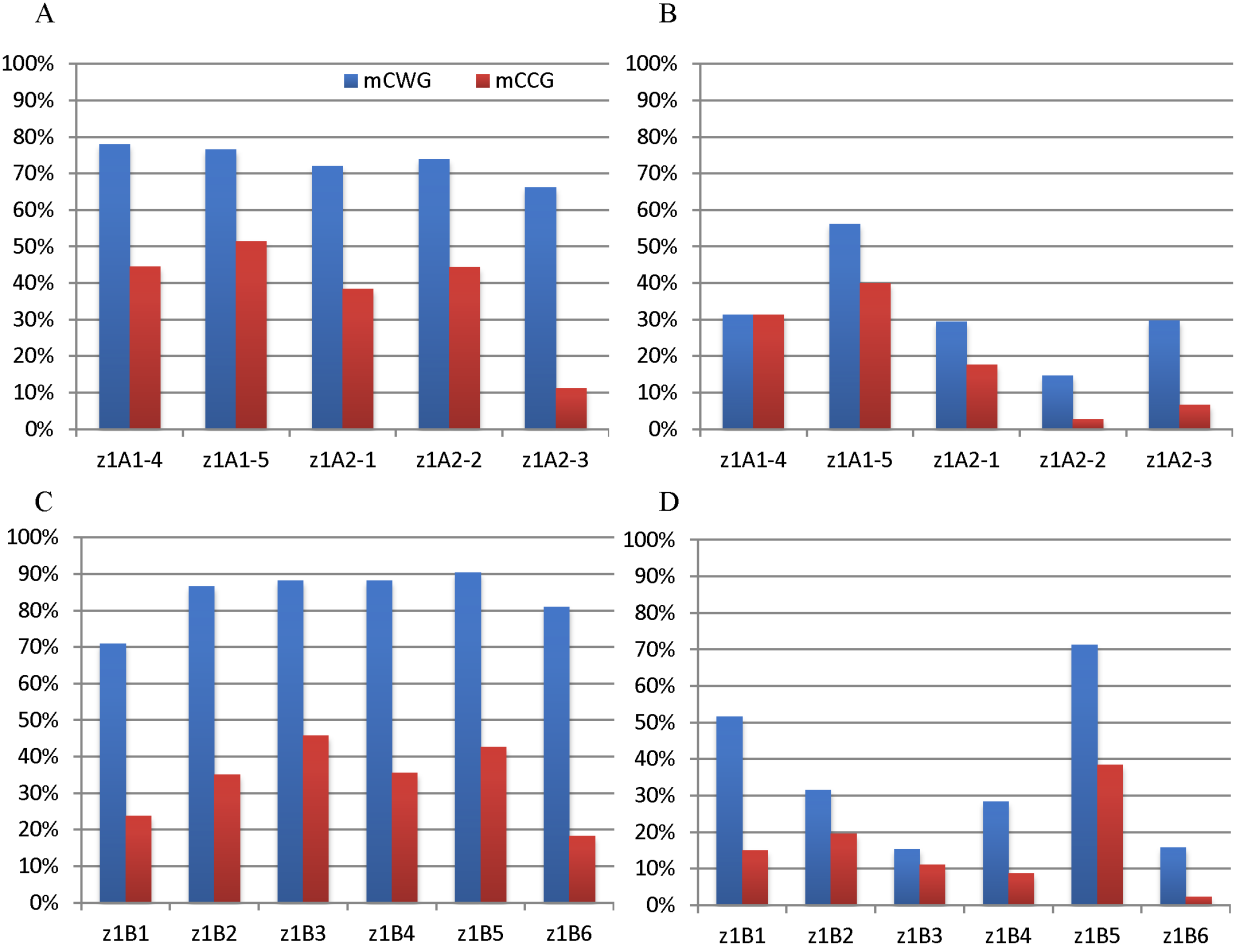
C methylation at mCWG and mCCG sites. Methylation levels of *z1A* (A, B) and *z1B* (C, D) gene coding regions in leaf (A, C) and endosperm (B, D) is shown. W represents A and T. The methylation of outer and inner C is marked with mCCG (blue) and CmCG (red), respectively.

DNA methylation of mCCG was also compared to mCWG (W = A and T). The average methylation of CWG was 70.87% and 51.55% in leaf and endosperm of the *z1B1* gene, respectively. The average of mCCG is 23.78% and 14.96%, which was three-fold lower than that of CWG. The average methylation of mCCG for *z1A* and *z1B* genes was 37.93% and 33.52% in leaf, 19.64% and 15.82% in endosperm, respectively, which was almost two-fold lower than the average methylation level of CWG (Fig 6, S2 Table), suggesting that mCCG methylation could be blocked by CmCG.

## Discussion

DNA methylation forms an important structural mark in chromatin that can be recognized by DNA-interacting proteins. In most of these cases DNA hypomethylation within promoter regions correlates with gene expression [8,9]. Previous studies also showed that zein and glutelin genes undergo specific and extensive undermethylation in endosperm [33]. In rice, tissue-specific expressed genes, encoding storage proteins and starch synthesizing enzymes, exhibited hypomethylated DNA in endosperm, indicating tissue specific DNA methylation levels during development. An interesting genetic phenomenon is paramutation, where a methylated epiallele can silence a paramutable allele in heterozygous progeny with the acquisition of methylated DNA. However in this case, methylation appears to be rather the consequence of gene silencing than the cause [34]. The same seems to occur after the activation of the autonomous element Activator (*Ac*), when tissue-cultured cells are regenerated to fertile plants, where DNA becomes demethylated as a consequence rather than a cause [23].

Tissue-specific expression of zein genes is in part achieved with two *trans*-acting factors Opaque2 and PBF that bind to two motifs in the promoter region, TCCACGTAGA and TAAAG, respectively [35,36]. The high CG methylation of TCCACGTAGA motif suppresses the expression of 22-kDa zein genes in leaf [30]. The promoters of *z1A* genes had high CG methylation in leaf, but lower non-CG methylation in leaf than in endosperm (Fig 1, S1 Table). This is consistent with *Arabidopsis* endosperm tissue, where DNA is hypomethylated only in the CG context whereas retaining high non-CG methylation [37]. However, rice endosperm has hypomethylated DNA in all three contexts [9]. In contrast to *z1A* genes, the promoters of *z1B* genes have similar DNA methylation patterns in both leaf and endosperm (Fig 3, S1 Table). Moreover, two expressed genes, *z1B4* and *z1B6*, have very low DNA methylation in their promoter regions in both leaf and endosperm, which suggests that DNA hypomethylation especially CG hypomethylation of promoter regions could contribute to gene expression levels.

Culturing plant tissue can alter the DNA methylation status even of TEs, which can be activated when plants are regenerated [21–23]. However, 19-kDa zein genes exhibited a different property, when endosperm tissue is cultured, which cannot be regenerated, but resembles gene expression in developing endosperm [38]. For *z1A* zeins, the average DNA methylation level of promoter regions in tissue-cultured endosperm is generally higher than normal leaf and endosperm, whereas the CG context is methylated at a medium level (Fig 1, S1 Table). Moreover, gene expression was not significantly reduced in *z1A* zein genes during tissue culture [30], whereas DNA methylation of the promoters of two highly expressed *z1B* zeins (*z1B4* and *z1B6*) was dramatically increased (Fig 3, S1 Table). In contrast, the DNA methylation of the lowly expressed gene *z1B1* was decreased at methylated cytosines in its promoter region of cultured endosperm when compared with normal tissues (Fig 3, S1 Table), suggesting that endosperm tissue culture could alter DNA methylation of *z1B* genes and activate their expression [30], which was consistent with previous studies in many plant species, such as maize [26,39,40], rice [41,42], barley [43], rye [44], tobacco [45] and carrot [27]. Whereas for *z1B* gene copies tissue culture could alter DNA methylation of the promoter region of genes and in particular increase the expression of the *z1B1* gene copy, this was not the case for *z1A* gene copies, thereby exhibiting locus-specific gene expression effects.

One interesting observation was that only the CHH context in the promoter regions of *z1B* zein genes was highly methylated at lowly expressed *z1B* gene copies in normal tissues, but for all *z1B* gene copies in cultured endosperm (S1 Table). Because methylation of CHH is controlled locus-specifically by the *DRM* and *CMT3* methyltransferase genes [46,47], this would suggest that *DRM* and *CMT3* genes control DNA methylation at the *z1B* locus.

In addition to the methylation status of promoter regions, the DNA methylation within gene bodies has also been implicated in plant gene expression [13,14,48]. In this case, genes methylated within their bodies are constitutively expressed at a higher level [8,9] and reduction of DNA methylation will lead to enhanced transcriptional levels [16]. In all 19-kDa zein genes, DNA methylation in endosperm was significantly lower than in leaf (Table 1), which is consistent with previous studies, where endosperm-specifically expressed genes that encode seed storage proteins and starch-synthesizing enzymes had lower methylation levels in endosperm throughout their coding regions [9,49,50]. Furthermore, CG and CHG methylation levels show more divergence in endosperm, indicating that endosperm tissue exhibits higher variations in methylation levels than other tissues (Table 1) [18]. Although studies had shown that DNA methylation levels within gene bodies correlated with transcription levels, it appeared to have little effect on 19-kDa zein gene expression levels. When comparing the two highest expressed *z1A2-1* and *z1B4* genes with others, they showed intermediate CHG methylation (28.64% in *z1A2-1* and 26.01% in *z1B4*) (Table 1). Although *z1A2-3* gene had similar CHG methylation (27.97%), it also has a premature stop codon. These results indicate that genes with intermediate CHG methylation (around 25%-30%) could still have high expression levels.

## Materials and Methods

### Plant materials

Leaf and endosperm harvested 18 days after pollination (DAP) from B73 and A636, and endosperm tissue culture from A636 were used for DNA extraction. The immature endosperms (13 days after pollination, DAP) of A636 were cultured on the semisolid MS medium, after the culture initiation of one to two months, calli were transferred into a liquid MS medium (without agar). Endosperm cells were cultured in the liquid suspension for more than one year with every 7 days sub-culturing. The tissues were cultured in the dark at 26°C and horizontal shakers at 160 rpm [38].

### Bisulfite sequencing

Genomic DNA of leaf, normal endosperm, and cultured endosperm was extracted following the CTAB protocol [51]. EpiTect Bisulfite Kit from Qiagen was used to conduct bisulfate conversions for genomic DNA following the manufacturer's instructions. Universal primers for the promoter of *z1A* and *z1B* zein genes are the same as in our previous study [30]. The other primers for the promoters and gene bodies of *z1A* and *z1B* zein genes were designed using Methyl Primer Express v1.0 (ABI) (S3 Table). DNA samples were amplified in an Eppendorf thermocycler, and PCR products were separated with a 1.8% agarose gel. Then, each purified sample was cloned into pGEM-T easy vector (Promega). Ninety-six colonies were picked and sequenced for each sample (leaf, endosperm and cultured endosperm) with each universal primer pairs. At least 10 colonies were picked and sequenced with each primer combinations.

### Sequence analysis

Each colony was sequenced with an ABI 3730XL DNA Analyzer at the Beijing Genome Institute (BGI, Shanghai). DNA sequences were aligned with the SeqMan program, and each 19-kDa zein gene copy was used as the reference to scan for all the cytosines. All cytosine sites were calculated based on conversion (C to T) rates (Cr), whereas cytosine methylation levels were 1-Cr.

## Supporting Information

S1 Fig The chromosomal location and polarity of 19-kDa zein genes. z1A comprises 12 genes in two locations on chromosome 4s and z1B has nine gene copies in one location on chromosome 7s, zein genes are presented as block arrows, and the red ones are the copies used for bisulfite sequencing analysis.
(PPTX)

S1 Table DNA methylation of 19-kDa zein gene promoters. (DOC)

S2 Table Methylation patterns of CmCG, mCCG and mCWG in *z1A* and *z1B* zein gene bodies. (DOC)

S3 Table Degenerate primers of 19-kDa zein genes for Bisulfite sequencing PCR amplification. (DOC)
