# Supplementary material for: Locus- and Site-Specific DNA Methylation of 19 kDa Zein Genes in Maize
Source: PLoS One. 2016 Jan 7;11(1):e0146416. doi: 10.1371/journal.pone.0146416 (PMC4704816; doi:10.1371/journal.pone.0146416)
Supplement: S1 Fig — z1A comprises 12 genes in two locations on chromosome 4s and z1B has nine gene copies in one location on chromosome 7s, zein genes are presented as block arrows, and the red ones are the copies used for bisulfite sequencing analysis. (PPTX) [file pone.0146416.s001.pptx]

## Slide 1
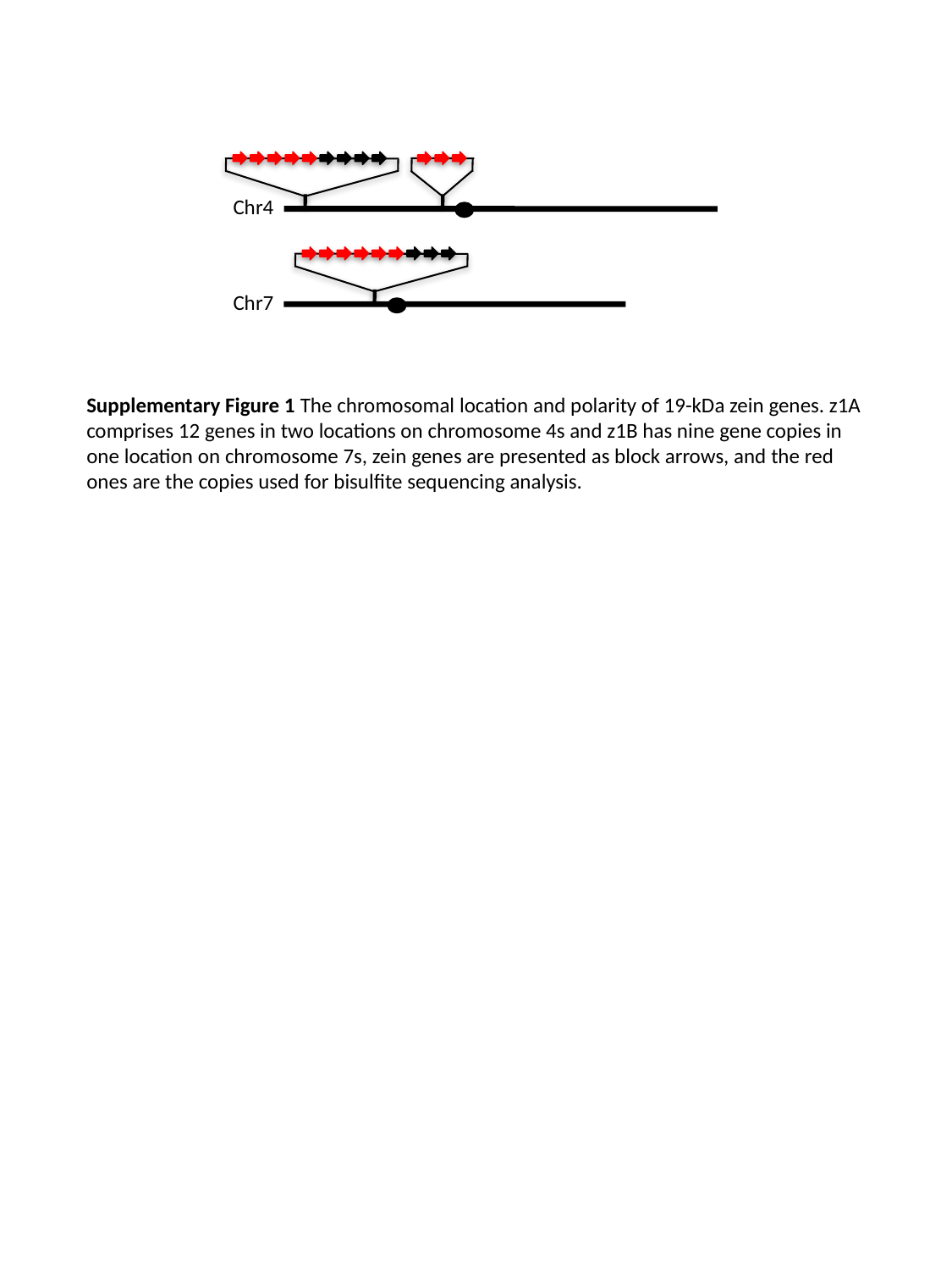

Chr4
Chr7
Supplementary Figure 1 The chromosomal location and polarity of 19-kDa zein genes. z1A comprises 12 genes in two locations on chromosome 4s and z1B has nine gene copies in one location on chromosome 7s, zein genes are presented as block arrows, and the red ones are the copies used for bisulfite sequencing analysis.
