# Supplementary material for: Locus- and Site-Specific DNA Methylation of 19 kDa Zein Genes in Maize
Source: PLoS One. 2016 Jan 7;11(1):e0146416. doi: 10.1371/journal.pone.0146416 (PMC4704816; doi:10.1371/journal.pone.0146416)
Supplement: S1 Table — (DOC) [file pone.0146416.s002.doc]

**Supplementary Table 1** DNA methylation of 19-kDa zein gene promoters

| Zeins |  | Average |  |  |  | CG |  | Expression ^b^ |
| --- | --- | --- | --- | --- | --- | --- | --- | --- |
|  | Leaf | Endosperm | TC |  | Leaf | Endosperm | TC |  |
| *z1A1-1* | 6.24%, 15^a^ | 3.85%, 10 | 7.08%, 10 |  | 71.23% | 26.67% | 30.00% | 0% |
| *z1A1-2* | 8.41%, 20 | 21.01%, 9 | 43.77%, 10 |  | 84.00% | 7.50% | 34.00% | 0% |
| *z1A1-3* | 8.06%, 14 | 2.57%, 20 | 55.71%, 9 |  | 81.46% | 13.00% | 80.00% | 0.69% |
| *z1A1-4* | 9.42%, 9 | 33.27%, 21 | 38.20%, 15 |  | 77.50% | 28.20% | 43.74% | 0.69% |
| *z1A2-1* | 2.64%, 16 | 3.19%, 15 | 30.89%, 11 |  | 98.20% | 41.63% | 55.55% | 96.56% |
| *z1A2-2* | 7.08%, 15 | 16.12%, 16 | 41.79%, 12 |  | 80.58% | 25.00% | 59.85% | 0.34% |
| *z1A2-3* | 8.32%, 18 | 4.21%, 18 | 25.65%, 9 |  | 89.32% | 11.12% | 42.00% | 0.34% |
| *z1B1* | 60.00%, 18 | 55.00%, 23 | 53.93%, 20 |  | 100% | 100% | 100% | 6.02% |
| *z1B2* | 65.00%, 17 | 73.06%, 12 | 56.90%, 8 |  | - | - | - | 0% |
| *z1B4* | 2.04%, 15 | 0%, 9 | 76.18%, 11 |  | - | - | - | 80.72% |
| *z1B5* | 42.25%, 10 | 20.96%, 21 | 42.93%, 20 |  | 50.00% | 6.00% | 54.00% | 0% |
| *z1B6* | 0%, 12 | 0.35%, 9 | 56.32%, 9 |  | - | - | - | 10.04% |

^a^ The clone number that was used for bisulfite sequencing analysis.

^b^ The gene expression level for each zein copy was referred to Miclaus et al. (2011).
