# Supplementary material for: Locus- and Site-Specific DNA Methylation of 19 kDa Zein Genes in Maize
Source: PLoS One. 2016 Jan 7;11(1):e0146416. doi: 10.1371/journal.pone.0146416 (PMC4704816; doi:10.1371/journal.pone.0146416)
Supplement: S2 Table — (DOC) [file pone.0146416.s003.doc]

**Supplementary Table 2** Methylation patterns of CmCG, mCCG and mCWG in *z1A* and *z1B* zein gene bodies.

|  | | Leaf | | |  | Endosperm | | |
| --- | --- | --- | --- | --- | --- | --- | --- | --- |
|  |  | CmCG | mCCG | mCWG |  | CmCG | mCCG | mCWG |
| *z1A* | *z1A1-4* | 77.76% | 44.42% | 78.00% |  | 74.29% | 31.23% | 35.96% |
|  | *z1A1-5* | 100.00% | 51.45% | 76.60% |  | 77.78% | 40.00% | 56.06% |
|  | *z1A2-1* | 87.86% | 38.38% | 71.99% |  | 65.37% | 17.62% | 29.44% |
|  | *z1A2-2* | 93.27% | 44.21% | 73.86% |  | 51.45% | 4.11% | 14.67% |
|  | *z1A2-3* | 80.15% | 11.18% | 66.20% |  | 30.56% | 6.67% | 29.70% |
|  | Average | 87.81% | 37.93% | 73.33% |  | 59.89% | 19.93% | 33.17% |
| *z1B* | *z1B1* | 78.46% | 23.78% | 70.87% |  | 88.00% | 14.96% | 51.55% |
|  | *z1B2* | 90.43% | 32.81% | 86.56% |  | 73.43% | 19.60% | 33.49% |
|  | *z1B3* | 95.83% | 45.77% | 88.22% |  | 56.66% | 11.01% | 15.33% |
|  | *z1B4* | 88.89% | 35.56% | 88.14% |  | 71.33% | 8.67% | 28.35% |
|  | *z1B5* | 97.43% | 42.63% | 90.37% |  | 97.43% | 38.40% | 71.31% |
|  | *z1B6* | 86.67% | 18.33% | 80.93% |  | 68.24% | 2.27% | 15.76% |
|  | Average | 89.62% | 33.15% | 84.18% |  | 75.85% | 15.82% | 35.96% |

W represents A and T.
