# Supplementary material for: Locus- and Site-Specific DNA Methylation of 19 kDa Zein Genes in Maize
Source: PLoS One. 2016 Jan 7;11(1):e0146416. doi: 10.1371/journal.pone.0146416 (PMC4704816; doi:10.1371/journal.pone.0146416)
Supplement: S3 Table — (DOC) [file pone.0146416.s004.doc]

**Supplementary Table 3** Degenerate primers of 19-kDa zein genes for Bisulfite sequencing PCR amplification

| Primers | Sequences (5’-3’) | Primers | Sequences (5’-3’) | Product size（bp） |
| --- | --- | --- | --- | --- |
| Bz1A_F | AGTGATTTTTTAAATYGATTATTAT | Bz1A_R | TATTTATACACATATCAATCCTTATACTT | 380-385 |
| Bz1B_F | TATGTGGTTAATGTTATATATGTGTAA | Bz1B_R | TTATTACTACTAAATTCCACTTTCTATATT | 349-350 |
| Bz1A2_F | ATTTTTTAAAYYGATTATTATATAAG | Bz1A2_R | CTACTAATCTAAGAGATATTTATACACATA | 384 |
| Bz1A2-3_F | TATTAAAATTAATATTGGGTTA | Bz1A_R | TATTTATACACATATCAATCCTTATACTT | 336 |
| 1/F | GTAGTAATAATAGAGTAATWATGG | 1/R | TACCAATAACTACACCAAAAATAAAA | 280-281 |
| 2/F | TTTATTTTTGGTGTAGTTATTGG | 2/R | TTTAACCAATTGGYCGCACTGAA | 258-261 |
| 3/F | ATTTAATTAATTGGTYGTATTGAA | 3/R | AACAATCTAAAAAADAACACC | 256-259 |
| 4/F | TTAATGTTTTTTTTGTAAAATTTAAAA | 4/R | TAAATTTTCACAYAYTAAAAACACC | 301 |
| 5/F | TTGGTTTTTTTGTAAGTGYTG | 5/R | AACAACAAAATTCAATACAACC | 353 |
| 6/F | GGTGTTTTYARTRTGTGAAAATTT | 6/R | CAACAAAACTCACAYYAACTAA | 413 |
| 7/F | AATTRGTARTATTGAATTTTGTTG | 7/R | TTCTAAAAMACCACAAACATCA | 307 |
| 8/F | TTGTATAAGTATAAGGATTGAGATGTG | 8/R | TACCAATAAATACACCAAAAATAACT | 388 |
| 9/F | GTTTTYGGTATGTGAAAATTTAAT | 9/R | CAAATAAAAAACAAAATTCAATACAA | 262 |
| 10/F | GTTATTTTTGGTGTATTTATTGGTA | 10/R | TAACAAAACTCACACCAACTAAC | 296 |
| 11/F | TGAATTTTGTTNTTTATTTGTAAT | 11/R | TTTCTTATTTCTAAAAAACCACA | 403 |
| 12/F | ATTGAGATGTGTATAAATATTTTTTAA | 12/R | CAATAAATACACCAAAAATAACTAC | 369 |
| 13/F | TGTATTGAATTTTGTTTTTTATTTG | 13/R | TTTCTAAAAAACCACAAACATCAAA | 400 |
| 14/F | AGTGATTTTTTAAATCGATTATTAT | 14/R | ACATAAATAACAATAATATAATATCC | 164 |
| 15/F | TGGTTATCGTTATATATGTGTAAAGGA | 15/R | TATTTATACACATATCAATCCTTATACTT | 262 |
| 16/F | TATCGTTATATATGTGTAAAGGTATTG | 16/R | TTAATTTTAAATTTTACAAAAAAAACA | 223 |

Primers Bz1A_F, R and Bz1B_F, R were the universal primers for z1A and z1B zeins, respectively (Miclaus et al. 2011). Bz1A2_F, R pair was used for z1A2 zein genes, and Bz1A2-3_F, Bz1A_R pair was used for z1A2-3 zein. Primers 1-3 for *z1B* gene body, Primers 4-7 for *z1A2* gene body, Primers 8-11 for *z1A1-4* gene body, Primers 9, 12 and 13 for *z1A1-5* gene body, Primers 14 and 15 for *z1A2-1* promoter, Primers 16 for *z1A2-2* promoter.
